# Diagnostic Accuracy of Fine Needle Aspiration Cytology in Parotid Lesions

**DOI:** 10.5402/2011/721525

**Published:** 2011-05-31

**Authors:** Naeem Sultan Ali, Shabbir Akhtar, Montasir Junaid, Sohail Awan, Kanwal Aftab

**Affiliations:** ^1^Division of Otorhinolaryngology-Head and Neck Surgery, Aga Khan University Hospital, Karachi 74800, Pakistan; ^2^Department of Pathology, Aga Khan University Hospital, Karachi 74800, Pakistan

## Abstract

*Objective.* Histopathology of parotid gland tumors is extremely varied and complex due to heterogeneous cellular composition. Preoperative diagnostic tools include fine needle aspiration cytology, the role of which remains controversial. The aim of this paper is to evaluate the usefulness and accuracy of fine needle aspiration cytology (FNAC) in the diagnosis of parotid gland tumors. *Methods.* We retrospectively reviewed charts of 129 patients who underwent parotidectomy for parotid lesions at Aga Khan University Hospital from 2002 to 2010. We compared the results of preoperative FNAC with final histopathological diagnosis. *Results.* Concordance with histological results was observed in 86%, specificity was 98%, sensitivity was 84%, and diagnostic accuracy was 94%. *Conclusion.* Our results demonstrate that preoperative cytology in parotid lesions is fairly accurate and useful in diagnosing benign from malignant and in planning appropriate approach for treatment.

## 1. Introduction

A lump in the salivary gland region often presents as a diagnostic challenge with regards to its site of origin, benign or malignant. Most of these occur in the parotid glands; small percentages occur in the submandibular, sublingual, and minor salivary glands. Parotid gland lesions are a histologically diverse group. Tumors of this region comprise 3% of all head and neck and 0.6% of all tumors of human body [1].

The history of fine needle aspiration cytology (FNAC) goes back to the 1920s where it came into use simultaneously in Europe and the United states [2, 3]. FNAC is a cytodiagnostic method based on the morphological findings of individual cells, group of cells, and microparticles of tissue, acquired using a needle. The role of FNAC for the diagnosis of salivary gland masses is well documented. The traditional open biopsy is no longer justified because of the risk of tumor spillage and damage to the facial nerve [4]. The method has a high degree of sensitivity in distinguishing the tumors from nonneoplastic lesions of salivary gland [5, 6].

FNAC is an easily done procedure with minimal incidence of complication and no risk of implantation of tumor cells (<1%). The complications are rare and bleeding or inflammatory reaction in the region of the puncture. The impairment of the involved nerves has been reported as a very rare complication [7, 8]. FNAC has its proponents and opponents. It is regarded as a diagnostic procedure to be superior to the combination of physical examination and radiological evaluation [9, 10]. Many authors claimed that it is accurate, safe and cost effective [11–13]. It can also be performed in children older than seven years [14]. However, Batsakis and colleagues were of the opinion that preoperative FNAC has little influence on the clinical management since most of the parotid masses ultimately require surgery [15]. This point of view ignores a considerable number of benign salivary tumors that do not necessitate surgery.

Because of the distinct morphology of parotid gland, the effectiveness of FNAC in its interpretation is still considered controversial. However, the use of FNAC helps to distinguish between reactive inflammatory process, which may not require surgery, and neoplastic lesions, between benign and malignant thus allowing proper planning before embarking any treatment. Surgical planning is dependent on clinical and radiological investigations; however, if the pathology is known preoperatively it is easy to counsel the patient and plan surgery. It is here where FNAC is of help specially for malignant lesions. In this article we describe our experience of using FNAC in the preoperative evaluation of parotid lesions.

## 2. Material and Methods

A retrospective review was carried out of medical records of patients that underwent parotidectomy for various pathologies between 2002 to 2010. A total of 205 parotid surgeries were performed during this period. Of these, only 129 patients were included in this study in which preoperative FNAC was done at our institution exclusively. Rest of the patients, in which we only performed surgery but FNAC was not carried out at our centre, were excluded.

FNAC was performed at our pathology department using a 22-gauge needle attached to a 10 mL syringe holder by a free hand technique. A minimum of two needle passes were made in each case. The specimens were expelled onto two or three slides, and thin smears were prepared between two slides and immediately fixed. The slides were generally stained with Papanicolaou and occasionally with May-Grunwald Giemsa methods. None of the FNAC was carried out with ultrasonography.

We classified our FNAC results into the following categories: true-negative (absence of malignancy correctly diagnosed), true-positive (presence of malignancy correctly diagnosed), false-negative (cytological specimen failed to diagnose a malignancy), and false-positive (cytological specimen was incorrectly considered or suspect for malignancy). 

We also compared the histopathology of the surgical specimens with the preoperative cytology of the FNAC specimens and evaluated the sensitivity, specificity, positive predictive value (PPV), negative predictive value (NPV), and overall accuracy of FNAC to differentiate between benign and malignant disease.

## 3. Results

There were 73 males and 56 females patients. The age range was 15 to 78 years with a mean age of 44 years. The final histological diagnoses of the included cases are listed in Table 1.

There were 98 benign lesions (89 neoplastic and 9 nonneoplastic) while 31 (24%) were malignant tumors (Table 2). Pleomorphic adenoma was the most common in benign tumor group (60%) while mucoepidermoid carcinoma (12%) in the malignant group. The FNAC smears were nondiagnostic in 5 (3.8%) cases, of which 4 were reported benign (2 neoplastic and 2 nonneoplastic) and 1 malignant lesion on final histopathology.

An overall of eighty-five percent (110/129) concordance between FNAC and final histological diagnosis was established, 78% with malignant and 88% with benign cases on break up (Table 2). The cytological diagnosis was true-positive in 26 (20%) cases and true-negative in 96 (74%) cases. Twenty-four of the 26 (92%) true-positive neoplasms and 86 of the 96 (90%) true-negative benign lesions were determined as an accurate results (Tables 3 and 4). There were 2 (1.5%) false positive and 5 (3.8%) false negative results (Table 5). 

A statistical analysis of 129 cases was carried out to assess the diagnostic accuracy of parotid FNAC compared with that of histological results (Table 6). A sensitivity of 84% was observed. The specificity was 98%, and the diagnostic accuracy was 94%. The positive and negative predictive values were 93% and 95%, respectively.

Local inflammation was observed in only two cases after performing FNAC; however, no complication such as hematoma, infection, or facial nerve damage was observed.

## 4. Discussion

The FNAC has been widely used as a diagnostic tool for the management of various head and neck lesions [16]. Many authors considered FNAC a superior modality and claimed it accurate and safe [6, 11–13]. In contrast, others argued that it has little influence on clinical management because of its high rates of false-positives and false-negatives and also ultimately patients have to undergo surgery [15]. However, the preoperative cytology helps to the differential diagnosis between benign and malignant lesions of parotid gland, and thus the extent of the surgery can be planned and modified accordingly.

The diagnostic sensitivity of cytopathology in detecting malignant disease was 84% in this study. This result implicates that if FNAC is used as a screening tool in this patient group, 12% of the malignant lesions would have been missed. These values fall within wide range of sensitivity reported in various studies, from as low as 27% up to 97% [9, 16–21]. The reason often cited for this wide range is the dependence on skills of the cytotechnologist performing FNAC and the expertise of the pathologist to assess adequacy and accurate examination of the specimen.

Specificity in our study was 98%. Specificity reported in the literature has been similarly high, in the range of 84% to 100% [9, 16]. The false negative FNAC results included a variety of lesions. A common reason for false negative FNAC findings is sampling error.

In the recent literature, the accuracy has ranges from 84% to 97% [9, 16, 22]. In our study it was 94%. The major drawback of the FNAC is the occurrence of nondiagnostic aspirations, which occur in 5 to 15% of cases in literature. In our study, only 5 (3.8%) aspirates were found to be non-diagnostic results. Failure to obtain a representative smear could be the result of needle positioning outside the target tissue or of necrosis, hemorrhage, or cystic areas in the tumor. In order to decrease chances of these errors and to increase the diagnostic accuracy various authors [23, 24] have utilized ultrasonography to assist FNAC but in our series it was not used. 

Because of the varied, complex and overlapping morphological features, the diagnosis of pleomorphic, monomorphic adenoma, and adenoid cystic carcinoma can be sometimes problematic. We had one case of monomorphic adenoma which was reported pleomorphic on FNAC. Similarly a case of carcinosarcoma was labelled as Warthin's tumor by FNAC (Table 5). The rate of false negative results was 3.8% in our review which is significantly less than what Zurrida et al. [9] and others have reported [16, 25]. Two pleomorphic adenoma were misinterpreted by FNAC and diagnosed as mucoepidermoid and myoepithelial carcinoma. This could have happened because of lack of typical features and the presence of atypical squamous cells on FNAC [26]. 

We correctly typed pleomorphic adenoma in 73 of 77 (95%) cases, better than the reported ranges from 82 to 94% in the literature [9, 27]. Mucoepidermoid carcinoma, according to Cohen et al. [28] is one of the most challenging lesions to diagnose and type cytologically. However, in our series 14 out of 16 were correctly classified. The two acinic cell and 4 adenoid cystic carcinoma were also correctly recognized in our series.

According to the experience of Shaha et al. [29] the diagnosis of the malignant lymphoma using the aspirated cytology is possible. Zurrida et al. [9] reported correct identification of only 2 of 7 cases of parotid lymphoma by FNAC while in a series of Al-Khafaji et al. [30], all ten lymphomas were accurately identified. In our study we were able to diagnose correct only 1 lymphoma of two cases. The fact that it was not detected as malignant on FNAC is consistent with other reports that lymphoma is difficult to diagnose by FNAC [31] and if a lymphoma is suspected on clinical grounds, flow cytometry of FNAC can be an adjunct in diagnosing it.

Still, to some authors, role of FNAC in the diagnosis of parotid lesions has not been well taken [32]. The only relative contraindication of the performing the FNAC could be the hemorrhagic disease. Many authors exclude the possibility of the implantation of the malignant cells or its recurrence caused by FNAB [33]. The phenomenon of tumor cells seeding has become a rare complication with the current use of small-bored needles [34]. We only had two cases of FNAC postprocedure local inflammation which resolved subsequently. No other complications such as hematoma, nerve damage, or infection was observed in our series.

The most important questions to be answered by this study is whether results gained from FNAC can be useful in the clinical management of patient with parotid lesions. Our experience has demonstrated a variety of circumstances in which such data may be valuable. The usual recommendation for the neoplastic lesions regardless of the preoperative cytological diagnosis is excision but recognition of benign lesion, beforehand like in case of Warthin's tumor, in poor risk patients may be of benefit in avoiding inappropriate surgery.

## 5. Conclusion

Our study shows that preoperative FNAC plays an important role in the accurate diagnosis of parotid tumors. It is a safe and effective modality for the treatment of patients with parotid lesion. This office based procedure is reliable, well tolerated, easy to perform and cost effective. Moreover preoperative differentiation of tumors may help prepare both the surgeon and patient for an appropriate surgical procedure.

## Figures and Tables

**Table 1:** Histological diagnosis.

Pleomorphic adenoma	77
Warthin's tumor	11
Sialadenitis	6
Monomorphic adenoma	1
Tuberculosis	1
Cyst	1
Lymph nodes	1
Myoepithelial carcinoma	1
Squamous cell carcinoma	1
Salivary duct carcinoma	1
Carcinosarcoma	1
Metastatic melanoma	1
Mucoepidermoid carcinoma	16
Adenoid cystic carcinoma	4
Acinic cell carcinoma	3
Lymphoma	2
Malignant mixed tumor	1

Total	129

**Table 2:** Histological and cytological diagnosis.

	Histology	Cytology discordant
Pleomorphic adenoma	77	4 (5%)
Warthin's tumors	11	2 (2%)
Sialadenitis	6	3 (50%)
Monomorphic adenoma	1	1
Tuberculosis	1	1
Cyst	1	
Lymph nodes	1	1
Total (Benign)	98 (76%)	12 (12%)

Myoepithelial carcinoma	1	
Sq cell carcinoma	1	
Salivary duct carcinoma	1	1
Carcinosarcoma	1	1
Metastatic melanoma	1	
Mucoepidermoid carcinoma	16	2 (1%)
Adenoid cystic carcinoma	4	
Acinic cell carcinoma	3	1
Lymphoma	2	1 (50%)
Malignant mixed tumor	1	1
Total (Malignant)	31 (24%)	7 (22%)

**Total**	**129**	**19 ( 14%)**

**Table 3:** True-positive with accurate and inaccurate results *n* = 26.

True positive (*n* = 26)			
Accurate (*n* = 24)		Inaccurate (*n* = 2)	
Cytologic diagnosis	Histologic diagnosis	Cytologic diagnosis	Histologic diagnosis
14 Mucoepidermoid	14 Mucoepidermoid	1 Myoepithelial ca.	1 Salivary duct ca.
4 Adenoid cystic ca.	4 Adenoid cystic ca.	1 Mucoepidermoid ca.	1 Acinic cell ca.
2 Acinic cell ca.	2 Acinic cell ca.		
1 Myoepithelial ca.	1 Myoepithelial ca.		
1 Squamous cell ca.	1 Squamous cell ca.		
1 Lymphoma	1 Lymphoma		
1 Metastatic melanoma	1 Metastatic melanoma		

**Table 4:** True-negative with accurate and inaccurate results *n* = 96.

True-negative (*n* = 96)			
Accurate (*n* = 86)		Inaccurate (*n* = 10)	
Cytologic diagnosis	Histologic diagnosis	Cytologic diagnosis	Histologic diagnosis
73 Pleomorphic adenoma	73 Pleomorphic adenoma	3 Pleomorphic adenoma	1 Sialadenitis
9 Warthin's tumor	9 Warthin's tumor		1 TB
1 Cyst	1 Cyst		1 Monomorphic adenoma
3 Sialadenitis	3 Sialadenitis	2 Lymphadenitis	1 Pleomorphic adenoma
			1 Lymph nodes
		4 Non-neoplastic	2 Sialadenitis
			1 Warthin's tumor
			1 Pleomorphic adenoma
		1 Sialadenitis	1 Warthin's tumor

**Table 5:** False Positive and Negative results.

False-negative (*n* = 5)		False-positive (*n* = 2)	
Cytologic diagnosis	Histologic diagnosis	Cytologic diagnosis	Histologic diagnosis
1 Pleomorphic adenoma	1 Malignant mixed tumor	1 Myoepithelial ca.	1 Pleomorphic adenoma
1 Cyst	1 Mucoepidermoid ca.	1 Mucoepidermoid ca.	1 Pleomorphic adenoma
1 Warthin's tumor	1 Carcinosarcoma		
1 Non-neoplastic	1 Mucoepidermoid ca.		
1 Lymphadenitis	1 Lymphoma		

**Table 6:** Comparison of histological results in 129 cases with preoperative cytology results.

		Histological diagnosis		
		Benign	Malignant	Total
FNAC Diagnosis	Benign	96 TN	5 FN	101
	Malignant	2 FP	26 TP	28

Total		98	31	129

TN = true-negative, TP = true positive, FN = false negative, and FP = false positive.
